# Evaluation of neonatally-induced mild diabetes in rats: Maternal and fetal repercussions

**DOI:** 10.1186/1758-5996-2-37

**Published:** 2010-06-08

**Authors:** Isabela L Iessi, Aline Bueno, Yuri K Sinzato, Kristin N Taylor, Marilza VC Rudge, Débora C Damasceno

**Affiliations:** 1 Botucatu Medical School, UNESP - Univ Estadual Paulista, Department of Gynecology and Obstetrics, Laboratory of Experimental Research in Gynecology and Obstetrics, São Paulo State, Brazil; 2 Weill Cornell Medical College, New York, USA

## Abstract

Many experimental studies have been performed to evaluate mild diabetes effects. However, results are divergent regarding glycemia and insulin measurement, fetal macrossomia, and placental weights. The aim was to investigate repercussions of neonatally-induced mild diabetes on the maternal organism and presence of congenital defects in their offspring in other mild diabetes model. On the day of birth, female offspring were distributed into two groups: Group streptozotocin (STZ): received 100 mg STZ/kg body weight, and Control Group: received vehicle in a similar time period. Maternal weights and glycemias were determined at days 0, 7, 14 and 21 of pregnancy. At day 21 of pregnancy, the rats were anesthetized and a laparotomy was performed to weigh and analyze living fetuses and placentas. The fetuses were classified as small (SPA), appropriate (APA) and large (LPA) for pregnancy age. Fetuses were also analyzed for the presence of external anomalies and processed for skeletal anomaly and ossification sites analysis. Statistical significance was considered as p < 0.05. In STZ group, there was increased glycemia at 0 and 14 days of pregnancy, lower weights throughout pregnancy, higher placental weight and index, an increased proportion of fetuses classified as SPA and LPA, and their fetuses presented with an increased frequency of abnormal sternebra, and absent cervical nuclei, which were not enough to cause the emergence of skeletal anomalies. Thus, this study shows that mild diabetes altered fetal development, characterized by intrauterine growth restriction. Further, the reached glycemia does not lead to any major congenital defects in the fetuses of streptozotocin-induced mild diabetic rats.

## Introduction

*Diabetes mellitus *(DM) is a metabolic disorder characterized by hyperglycemia, insufficient insulin secretion, and receptor insensitivity to endogenous insulin. Its incidence is associated with high morbidity and mortality rates [1]. In pregnancies complicated by diabetes, hyperglycemia and alterations in lipid metabolism are associated with both maternal and fetal complications [2,3], causing reproductive abnormalities that enhance spontaneous abortion, congenital anomalies, and neonatal morbidity and mortality [4,5].

Congenital anomalies are more common in infants of diabetic women than in children of nondiabetic women. The etiology, pathogenesis and prevention of diabetes-induced anomalies have spurred considerable clinical and basic research efforts. The infant of the diabetic mother also has increased risk for several neonatal complications, such as macrosomia, hypoglycemia, hypocalcemia, polycythemia and hyperbilirubinemia. Up to 25% of such offspring have been reported with these complications. It also appears that early detection and subsequent strict metabolic control of pregnant women with diabetes in pregnancy should decrease the frequency and severity of some of these short- and long-term complications in the offspring of the diabetic mother [4].

Despite increased clinical efforts to improve glycemic control during diabetic pregnancy, however, the rate of congenital malformations remains increased in studies of Diabetes mellitus (DM) of type 1 [6-9], DM type 2 [9-12], and gestational diabetes (GDM) [10,13]. The prevalence of major congenital malformations is approximately three to five times higher in infants of diabetic mothers [14-17] and is presently the most common cause of perinatal death among these infants [18,19]. Diabetes is associated with a variety of anomalies, primarily cardiovascular, neurological, and musculoskeletal [20]. The malformation considered to be most pathognomic to the infants of diabetic mothers - caudal regression syndrome or sacral agenesis - is claimed to be 200-400-fold more frequent [21] but is still a rare anomaly.

Studies in humans that explore the responsible mechanism for these alterations are limited not only by ethical reasons but also by the multiplicity of uncontrolled variables that may modify the intrauterine environment and cause potential effects on congenital malformations. Therefore, there is a need for appropriate animal models [22].

In order to reproduce the clinical status of uncontrolled type 1 DM, experimental models are used to obtain severe diabetes (glycemia > 300 mg/dL) [23-25]. The complications that affect in mother and fetus that result from this model are well-known. Besides, other models were developed in laboratory animals to reproduce the clinical conditions of the DM type 2 and GDM. Similarly, the experimental model proposed is identified as mild or moderate diabetes (glycemia between 120 and 300 mg/dL). To obtain this glycemic level, several methodologies may be used, such as administration of different doses of a beta-cytotoxic agent (streptozotocin) during the period neonatal [26,27] or streptozotocin injection during pregnancy [28-30]. However, many experimental studies have been performed to evaluate the effects of mild diabetes, with divergent results regarding glycemia and insulin measurement, presence of fetal macrosomia and placental weights. In our laboratory, two streptozotocin administration (day 1 of birth and at day 7 of pregnancy of Wistar rats) were performed and the results showed that this mild diabetes model led a negative impact on maternal reproductive performance and caused intrauterine growth restriction and impaired fetal development (in press). Due to negative effects of this diabetes model and controversial information of the mild diabetes effects about maternal and fetal repercussions. The present study aimed to investigate the repercussions of mild diabetes in the maternal organism and the presence of congenital defects in their offspring.

## Methods

Wistar rats were obtained from São Paulo State University (Unesp), Botucatu, São Paulo State, Brazil. They were maintained in an experimental room under controlled conditions of temperature (22 ± 2°C), humidity (50 ± 10%), and a 12 hour light/dark cycle. Protocols for animal use and procedures necessary for the experiments described here were approved through the Local Experimental Ethical Committee for Research, which assures adherence to the standards established by the Guide for the Care and Use of Laboratory Animals.

Parental non-diabetic female rats were mated with non-diabetic males to obtain newborns (NB). On day 0 of birth, female NB were distributed into two groups: diabetic group (STZ, n = 102) and in the STZ group female NB received a streptozotocin injection (STZ - Sigma Chemical Company, St. Louis, Millstone, USA) administered at a dose of 100 mg/kg (0.1 M, pH 4.5) subcutaneously (sc) according to modified procedures [26,27]; non-diabetic group (control, n = 45). In the control group, female NB received only citrate buffer administered sc. NB rats remained with their mothers until day 21 of life (weaning period). In adult life, the female rats were mated overnight with non-diabetic male rats. The morning when spermatozoa were found in the vaginal smear was designated gestational day (GD) 0. STZ rats presenting with glycemia between 120 and 300 mg/dL at GD 0 were characterized as having mild diabetes, and non-diabetic rats with glycemia below 119 mg/dL were considered control and were included in this study. On days 0, 7, 14 and 21 of pregnancy, maternal body weights and glycemia were determined. Blood glucose concentrations were measured by a One-Touch Ultra glucometer (LifeScan, Johnson and Johnson^®^). Values were expressed in mg/dL.

On day 21 of pregnancy, fed rats were anesthetized with sodium thiopental (50 mg/kg). Following trichotomy of the abdominal region, the animal was placed in the dorsal decubitus position, and its libs were fixed to the surgery table. The laparoscopy procedure was carried out by a midline incision beginning at the xiphoid cartilage and ending at the pubis. The intestinal loops were moved cranially for uterus exposure. Immediately following exploratory laparotomy, all viable fetuses and placentas were weighed for determination of placental index (placental weight/fetal weight). The fetuses were classified by the mean ± 1.7 SD according to the mean values of fetal weights of the control group: as small for pregnancy age (SPA) when weight was smaller than control mean - 1.7 SD; appropriate for pregnancy age (APA) when weight was included in control mean ± 1.7 SD; and large for pregnancy age (LPA) when weight was greater than control mean + 1.7 SD, and the data were presented as percentual values [3,31]. The fetuses were externally evaluated by microscope with respect to the incidence of external anomalies. After external analysis, half of the fetuses were prepared for skeletal examination by the staining procedure of Staples & Schnell [32]. In addition to the skeletal analyses, the counting of the ossification sites was performed according to methodology proposed by Aliverti [33], which determines the degree of fetal development. The remaining fetuses were prepared for visceral examination into another study in our laboratory (data not published).

The results were reported as mean ± standard error (SEM) or standard deviation (SD) of mean. All data were statistically analyzed using Student-Newman-Keuls test [34]. For fetal weight classification, the Chi-square test was used [35]. Statistical significance was considered as p < 0.05.

## Results

In the present study, 102 female rats had diabetes induced by STZ at the neonatal period. Of these, 82 rats reached adult life and were mated, and 76 of these presented with a positive vaginal smear. On GD 0, only 37 female rats presented with glycemia between 120 and 300 mg/dL and were included in the STZ group; only 28 rats reached term pregnancy. In the control group, 45 female rats received citrate buffer during the neonatal period. All of the rats reached the reproductive phase and were mated. Of the 45 mated female rats, 37 were pregnant with glycemia less than to 119 mg/dL and were included in control group. On day 21 of pregnancy, 28 rats had reached term pregnancy.

### 3.1 Glycemia during pregnancy

In control rats, normoglycemia was confirmed with mean glucose values below 119 mg/dL (day 0, 7, 14 and 21 of pregnancy). In STZ rats, a significantly higher glycemia (p < 0.05) was verified on days 0 and 14 of pregnancy compared to control rats (Table 1).

**Table 1:** Glycemic levels on days 0, 7, 14 and 21 of pregnancy in mildly diabetic (STZ) and non-diabetic (control) rats.

	Groups	
	**Control** **(n = 28)**	**STZ** **(n = 28)**

**Maternal glycemia (mg/dL)**		
**Day 0**	105.00 ± 1.81	131.70 ± 1.87*
**Day 7**	102.80 ± 2.36	106.10 ± 2.56
**Day 14**	83.10 ± 1.45	92.90 ± 2.45*
**Day 21**	85.30 ± 1.92	83.70 ± 2.69

Data are reported as mean ± standard error of mean (SEM)

*p < 0.05 - statistically significant difference compared to control group (Student-Newman-KeulsTest).

### 3.2 Maternal weight gain during pregnancy

Throughout pregnancy, an increase in body weight in STZ and control rats was observed. However, on days 0, 7, 14 and 21 of pregnancy, the STZ dams presented with lower body weight (p < 0.05) and maternal weight gain (day 21 - 0) compared to control rats (Table 2).

**Table 2:** Body weight on days 0, 7, 14, 21 and maternal weight gain (day 21 - 0) in pregnant mildly diabetic (STZ) and non-diabetic (control) rats.

	Groups	
	**Control** **(n = 28)**	**STZ** **(n = 28)**

**Day 0**	284.19 ± 4.66	260.71 ± 7.99*
**Day 7**	305.29 ± 5.09	278.81 ± 7.90*
**Day 14**	330.96 ± 5.36	303.26 ± 8.71*
**Day 21**	407.22 ± 6.43	357.86 ± 11.67*
**Maternal weight gain (day 21-0)**	123.03 ± 2.91	97.81 ± 4.91*

Data are reported as mean ± standard error of mean (SEM)

*p < 0.05 - statistically significant difference compared to control group (Student-Newman-KeulsTest).

### 3.3 Fetal weight, placental weight and placental index

There was no significant difference in fetal weight between STZ rats and control rats. STZ rats presented with higher placental weight and index (p < 0.05) relative to control group (Table 3).

**Table 3:** Fetal weight, placental weight and placental index in mildly diabetic (STZ) and non-diabetic (control) rats at term pregnancy.

	Groups	
	**Control** **(n = 28)**	**STZ** **(n = 28)**

Fetal weight	5.40 ± 0.05	5.34 ± 0.09
Placental weight	0.40 ± 0.01	0.46 ± 0.01*
Placental index	0.08 ± 0.00	0.09 ± 0.00*

Data are reported as mean ± standard error of mean (SEM)

*p < 0.05 - statistically significant difference compared to control group (Student-Newman-KeulsTest).

### 3.4 Classification of fetal body weight

**Figure 1:**
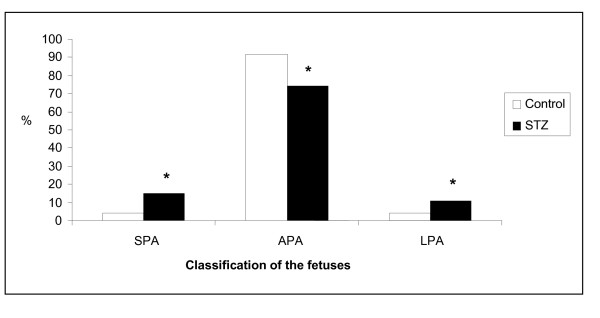
**Proportion (%) of fetuses classified as small (SPA), appropriate (APA) and large (LPA) for pregnancy age from mildly diabetic (STZ) and non-diabetic (control) rats at term pregnancy**. * p < 0.05 - statistically significant difference compared to control group (Chi Square Test).

The fetuses from the STZ group presented with a significant increase in the proportion of fetuses classified as small (SPA) and large (LPA) for pregnancy age, and a significant reduction in the proportion of APA (appropriate for pregnancy age) fetuses compared the from control group (Figure 1).

### 3.5 Analysis of the frequency of external and skeletal anomalies

Table 4 illustrates fetuses from STZ female rats that presented no significant difference in the rates of external anomalies compared to the control group. Fetuses from the STZ group presented with a higher number of abnormal sternebra and absent cervical nuclei (p < 0.05).

**Table 4:** Frequency of external and skeletal anomalies in fetuses from mildly diabetic (STZ) and non-diabetic (control) rats at term pregnancy.

	Groups	
	**Control** **(n= 15)**	**STZ** **(n= 13)**

Number of fetuses examined for external anomalies	198	138
Number of fetuses with external anomalies	0	0
Number of fetuses examined for skeletal anomalies	81 (100%)	55 (100%)
		
Abnormal shaped sternebrae	2/81 (2%)	5/55 (9%)*
Extra ossification site of sternebrae	0/81(0%)	1/55 (2%)
Incomplete ossification of vertebrae	1/81 (1%)	0/55 (0%)
Absent cervical nuclei	50/81 (62%)	57/55 (104%)*
Bipartite cervical nuclei	3/81 (4%)	1/55 (2%)
Incomplete ossification of cervical nuclei	5/81 (6%)	5/55 (9%)

*p < 0.05 - statistically significant difference compared to control group (Chi Square Test).

### 3.6 Ossification sites

There was no significant difference (p>0.05) in the number of ossification sites in fetuses from STZ group in relation to the control group (Table 5).

**Table 5:** Ossification sites in fetuses from mildly diabetic (STZ) and non-diabetic (control) rats at term pregnancy.

	Groups	
	**Control** **(n = 15)**	**STZ** **(n = 13)**

Sternebrae	6.00 ± 0.00	6.01 ± 0.05
Anterior phalange	3.97 ± 0.08	3.97 ± 0.27
Posterior phalange	2.57 ± 1.09	2.42 ± 1.08
Metacarpus	4.00 ± 0.00	4.03 ± 0.19
Metatarsus	4.97 ± 0.05	4.75 ± 0.33
Number of caudal vertebrae	4.38 ± 0.80	4.29 ± 0.89
Total number of ossification sites	25.88	25.52

p > 0.05 - non statistically significant difference

## Discussion

In the present study, it was verified that neonatally streptozotocin-induced diabetic rats presented glycemia superior than 120 mg/dL on day 0 of pregnancy, confirming the inclusion criterion for the diabetic group. On day 14 of pregnancy an increase in glycemic mean was also observed in this group, which was accompanied by glucose intolerance and insulin resistance (data submitted to publication - not shown in this paper) as demonstrated by the glucose and insulin tolerance tests, respectively, reproducing the clinical state of gestational diabetes that occurs at weeks 24-26 of human gestation [36,37]. The literature shows conflicting results about glycemia and insulin levels in the different models of mild diabetes induction. However, the experimental model that presented more convincing results (glycemia between 120 and 300 mg/dL) was the induction performed during the neonatal period [26,27,38,39].

Our results showed that STZ rats presented increased maternal weights since the beginning of pregnancy. There is a natural progressive increase in maternal weight during gestation due to fetal growth apart from adaptations of the maternal organism [40,41]. However, the weight gain of STZ rats during the pregnancy was lower compared to control group. The lower gain of maternal weight in the presence of mild diabetes may be related to STZ administration at neonatal period. There were early damages to the pancreatic β-cells, reducing insulin secretion which is considered major growth factor, contributing to impaired perinatal development and leading to consequences in the adult life of neonatally streptozotocin-induced diabetic rats, as also confirmed by Kim et al. (2006) [42]. These authors related the reduction of maternal weight gain to alterations in growth hormone (GH) and insulin-like growth factor (IGF-1) in the neonatally streptozotocin-induced diabetic female rats.

Infants of diabetic or prediabetic women frequently have increased birth weight and length [43]. They have also been found to have enlarged islets of Langerhans and β-cells [44] and higher than normal concentrations of plasma insulin. Further, there is evidence that insulin injection into rats during pregnancy results in larger than normal fetuses. Experimental models of mild diabetes induction (glycemia between 120 and 300 mg/dL) showed increased rates of macrosomic fetuses (LPA) [3,28,29]. In contrast with these studies, our study revealed increased rates of fetuses classified as small for pregnancy age in mildly diabetic dams. Similarly, Kervran *et al*. (1978) [45] did not obtain macrosomic fetuses in the offspring of rats with mild hyperglycemia during pregnancy. The difference between the findings in clinical and experimental studies might be justified by the relatively short pregnancy time in the rat, the difference in the percentage of adipose tissue in rat fetuses (1%) and human offspring (16%), and the greater weight gain in the human species. Our findings corroborate results found by Aerts & Van Assche (2006) [46] and Holemans *et al*. (2003) [47], which also observed fetuses with intrauterine growth restriction (IUGR) originated from mildly diabetic rats. An epidemiological relationship between low birth weight and impaired glucose tolerance in late life has been shown [48,49] and evidence for both insulin resistance and impaired function of the pancreatic β-cell in adulthood has been presented [50-52]. The human fetuses classified as small for gestational age (SGA) fetus is hypoinsulinemic and hypoglycemic [53,54]. A glucose challenge in the uterus provokes only a small insulin secretory response [55], and this inability persists in the neonate [56,57]. In the few cases which have been morphologically investigated, there is evidence of a reduced pancreatic β-cell mass [58]. This suggests reduced growth and impaired functional development of the insulin-producing β-cell in growth-retarded fetuses [49,50]. Fetal growth is a complex process that depends on the genotype and epigenotype of the fetus, maternal nutrition, the availability of nutrients and oxygen to the fetus, intrauterine insults, and a variety of growth factor and proteins of maternal, fetal and placental origin [59]. During the first trimester of pregnancy, embryonic growth might be controlled at the level of the individual organs by nutrient supply and by locally active growth factors. Later, fetal growth depends essentially upon materno-placental cooperation in delivering nutrients to the fetus. Fetal growth seems to be regulated by fetal insulin, insulin-like growth factor (IGF-1) and IGF-2, while growth hormone (GH) has only a secondary hole to play [60]. During pregnancy placental growth hormone (PGH) is the prime regulator of maternal serum IGF-1. Therefore the major hole of hormones in fetal growth is to mediate utilization of available substrate. The alterations in the maternal GH/IGF axis may lead to permanent pathological fetal programming of the IGF axis [59], causing late consequences of poor fetal environment reflected in intrauterine growth restriction, as confirmed by our results.

It was observed in our study that the placentas of mildly diabetic rats had a higher weight, which might represent a compensatory mechanism to assure the maternal-fetal exchanges contributing to fetal development. This justifies the similar mean fetal weights between the two groups (STZ and control). The increased weight of the placentas also contributed to increased placental index in the diabetic group. However, the increased placental weight/index was not enough to improve fetal development, as confirmed by increased rate of fetuses classified as small for gestational age.

Maternal diabetes during pregnancy is known to increase the risk of congenital malformation in offspring. The malformations associated with diabetic pregnancy affect many major organ systems, such as the central nervous, cardiovascular, gastrointestinal, urogenital and musculoskeletal systems [21,61]. The incidence of congenital malformations in diabetic pregnancies remains two-to six-fold higher than in non-diabetic pregnancies [8,18,19]. Although clinical studies have indicated that the risk for congenital malformations is dependent on blood glucose levels in early pregnancy, recent studies show that even in diabetic pregnant women with near optimal maternal glycemic control (HbA1c < 7%), the incidence of congenital malformations is still high [8,62].

In this investigation was verified that the rates analysis of external anomalies of the fetuses from mild diabetic rats presented no significant abnormalities related to mild maternal diabetes. It was observed that fetuses from neonatally streptozotocin-induced mild diabetic rat presented an increased frequency of abnormal sternebra and absent cervical nuclei. However, these skeletal abnormalities are not considered to be major anomalies. In other words, the alterations caused by mild diabetes were not sufficient to cause the emergence of skeletal anomalies. The number of ossification sites among the experimental groups showed that there was not somatic immaturity in the development of the fetuses of female rats with mild diabetes, showing that the glycemic intensity did not influence the studied variable. 	 In conclusion, mild diabetes caused alterations in fetal development characterized by intrauterine growth restriction, which was evidenced by the increase of the proportions of fetuses classified as small for pregnancy age. This glycemic intensity led no major congenital defects in the fetuses of streptozotocin-induced diabetic rats, thus it was associated no teratogenic effect in these fetuses.

## Competing interests

The authors declare that they have no competing interests.
